# Lithotomy

**Published:** 1829-02

**Authors:** 


### LITHOTOMY.

*Case of Lithotomy, (the subject of the Libel in the Lancet)*
By Bransby B. Cooper, Esq.

Stephen Pollard, æt. fifty-three, of a plethoric habit, but portraying want of constitutional power, admitted into Job's Ward, Guy's Hospital, March 7, 1828. He states that he has been subject to a gravelly deposit in his urine for seven years, and a twelvemonth after its first appearance he was attacked with excruciating pain in the region of the right kidney, which was constant and severe, and confined him to his bed for three months; at the end of which time he voided a stone with his urine, about the size of a barleycorn. Subsequent to this his health became re-established, suffering but a slight inconvenience from the sediment in his urine, which remained unaltered. In three years a second attack, similar to the first, took place on the opposite or left side; the same symptoms supervened, and at the end of a fortnight he voided another calculus, of nearly an equal size with the first. He soon recovered his health, and the gravelly sediment, though continuing, has latterly been much diminished in quantity. About a twelvemonth ago, unusual irritation in his bladder attracted his notice, which rapidly increased, causing a difficulty in micturition, the urine suddenly stopping, and the complete evacuation of the bladder inducing intense suffering. At length he was obliged to apply to a surgeon, who advised his coming to Guy's Hospital.

Upon his admission, he stated that his journey to town from Sussex, in a cart not hung on springs, gave him great uneasiness, producing frequent inclinations to void his urine. Walking also increases the symptoms. The pain is most considerable when the bladder is empty. The extremity of the prepuce is not much swollen, neither has he ever passed bloody urine. The sound being introduced, indicated the presence of a hard calculus. His general health not much impaired, but suffering from a slight catarrh, from exposure during his coming to London.

The operation was performed on Tuesday, the 18th of March. The sound being introduced, the calculus was felt with difficulty, and then only while withdrawing the instrument. The narrowness of the perineum excited attention. The straight staff being introduced, the external incision was purposely extended beyond the usual length, to compensate for the natural deformity. The groove of the staff was cut into, and the knife readily passed into the bladder, as indicated by the flow of a small quantity of urine. On passing my finger into the wound, the extent of the section of the prostate could not be ascertained, in consequence of the depth of the perineum; and, upon introducing the forceps, the stone could not be felt; I was, therefore, induced to enlarge the opening by means of Sir Astley Cooper's beaked knife. I then withdrew the straight staff, passed a curved one into the bladder, and detected the stone in the concavity of the curve, and, to secure the passage into the bladder, passed the cutting gorget (which was necessarily furnished with a beak), and used this as a guide to the introduction of the forceps; but still, though the forceps passed readily into the bladder, as was experienced by Mr. Callaway as well as myself, the stone eluded detection. A female staff was then passed into the wound, but could not be brought in contact with the stone. A male sound was next introduced through the incision into the bladder, and with some difficulty indicated the stone *above* the prostate, and consequently *behind* the pubes; and at length the blades of the forceps (the handles being directed downwards and backwards,) were brought in contact with the calculus, which, immediately on being felt, was extracted without any force, although, from the circumstances above detailed, the operation had unavoidably been tedious. When he was replaced in bed, he felt depressed and exhausted. Forty drops of laudanum were given, which produced slight composure, but no sleep.

Five o'clock.—Complains of very acute pain in the lower part of the abdomen, especially in the left iliac region; this increases on pressure. No tension of the abdomen is discernible.

**Apply thirty leeches, and hot fomentations.**

Ten o'clock.—The pulse has increased in number to 116, and is tremulous. The pain of the abdomen unrelieved by the leeches. The breathing is hurried, and the skin bedewed with a clammy perspiration. The countenance is natural. Answers questions with great composure.

**Ordered Hyd. Subm. gr. iij.; Ext. Opii gr. ij. M. to be taken directly. —A large emollient poultice to cover the whole of the abdomen.**

March 19th, one o'clock A.M.—Has not had any sleep. The tenderness of the abdomen undiminished; pulse 120, small, with a degree of hardness. For the last half hour has had nausea, and insufficient efforts to vomit, which greatly distress him, by increasing the pain.

**Repeat the calomel and opium.**

Five o'clock.—The pain in the abdomen is increased; the pulse 120, small, and hard; respiration difficult; nausea unabated.

**V.S. ad ℥x.**

This relieved the urgency of his symptoms, but was followed with depression.

**Ordered Hyd. Subm. gr. iij.; Opii Ext. gr. i. stat.—Cont^r Cataplasma.**

Ten o'clock.—The pain in abdomen continues; pulse as quick as in last report; tongue covered with a white fur, but moist; nausea still present, even rather more urgent. A sinapism ordered to be applied to the pit of the stomach, and thirty leeches to the abdomen. These gave immediate relief, to such an extent as to enable him to sleep.

One o'clock P.M.—Pulse 156, and irregular as to power, but constant in number. The anxiety of countenance indicates a fatal depression, and has a peculiar yellow hue, the lips being pale.

The nausea has returned, and the pain of the abdomen is only complained of during the spasm. The respiration is short, hurried, and attended with pain.

℞. Ammon. Carb. gr. iv.; Tr. Opii gtt. xxiv.; Infus. Serpent. ℥iss. fiat haust. to be taken directly.

After having taken this draught he slept two hours, when the respiration was twenty-six in a minute. He awoke in an alarming state of depression, the countenance anxious and pallid; he reluctantly answered questions, but said he was entirely free from pain. He took a small quantity of brandy-and-water, with the julep of ammonia, but continued gradually sinking until half past seven, when he died.

It may be worthy of remark, that this patient felt convinced in his own mind that the operation would prove fatal; and so strong was this impression, that he persuaded two patients in the same ward to show him the burial ground of the hospital. He visited this, and expressed his conviction that it would be *his* resting place.

*Examination of the body, sixty hours after death.* (From the notes of Dr. HODGKIN.)—The peritoneum, at the lower part of the abdomen, as well as that portion which lines the parietes and that covering the intestines, was minutely injected. In the pelvis there was some sero-sanguineous effusion, very slightly puriform, and unmixed with lymph or flocculi. Behind the peritoneum, in the posterior part of the left iliac region, there was some ecchymosis. The cellular membrane behind the peritoneum in the pelvis was extremely lacerable, readily breaking down under the finger, and scarcely requiring the use of the knife for the removal, except under the pubes. There was a free division of the prostate, and a clean cut into the bladder, the mucous membrane of which was generally healthy. Immediately behind the meatus urinarius there was a small tongue-shaped body, which, on the opening of the bladder, and when obscured by coagula, was considered to be the third lobe of the prostate; but a more careful examination proved it to be a small flap, composed of a portion of bladder and prostate,, and which had been formed by another incision communicating with the first, about an inch in length, and a third of an inch behind the opening of the meatus. There were a few spots of the ecchymosis and abrasion comprehended in a space of about the size of a shilling around the orifice of the meatus. The edges of the incision, from the external opening to the bladder, were ragged, and intermixed with adherent coagula of blood; a state which was unavoidably produced by the repeated introduction of the forceps and other instruments which were had recourse to in the attempt to remove the stone.

In the preparation, a passage exists at the side of the bladder. This was not noticed by Dr. Hodgkin till after it had been in the hands of the reporter of the Lancet; and, from the extremely lacerable state of the part, it might easily have been formed after its removal from the body. That it was either formed then or in the act of removing them, is an idea which the absence of coagula tends strongly to confirm.

Besides the injection of the peritoneal coat of the small intestines, the internal membrane was of a diffused red. The rectum was perfectly sound and healthy, with the exception of a very slight appearance of piles. The kidneys were of a moderate size, soft and flabby, and in an advanced stage of the light mottling deposit described by Dr. Bright.

This case resembles all those of unsuccessful lithotomy which I have myself had an opportunity of examining, both in the peritoneal inflammation and in the extensively lacerable state of the cellular membrane behind the peritoneum. Similar results have, I believe, invariably been found by Mr. C. A. Key in this country, and by my friend, Harvey de Chegoin, in Paris.

The peculiar derangement of the kidney observed in this case was likewise met with in a patient of Mr. C. A. Key's, who died after an operation for the stone; and has likewise been found in others who have sunk after the operation or accident.

---

*Case of Lithotomy, with unusual Difficulty in the Operation, at the* WINCHESTER HOSPITAL. By Mr. W. J. WICKHAM, Jun.

GEORGE LOCK, æt. four years, was admitted with calculus in the bladder into the Winchester County Hospital, November 12, 1828. The symptoms of stone had commenced when he was about fourteen months old; his health was otherwise good, but he was somewhat emaciated from continued and very great suffering.

Nov. 25th.—*Operation.*—The process of sounding being duly accomplished, and the existence of a stone having been distinctly ascertained, the operation proceeded as follows: By one plunge of the knife the first incision was effected, and the urethra opened near the prostate gland; the beak of the gorget was at once lodged in the groove of the staff, and passed onwards into the bladder. The arrival of the gorget in the bladder was not announced by a gush of urine, as it had been voided entirely on the introduction of the staff. I now passed my finger at once into the wound, and felt the stone at the fore and upper part of the bladder, towards the pubes. I then introduced the forceps, and felt the stone in the situation I had found it with my finger; but it was not bared, a substance evidently intervening between the forceps and the stone. I withdrew the forceps, and again passed my finger, but did not feel the stone exposed. At the moment I conceived that the forceps, and my finger on its second introduction, had found their way between the bladder and the rectum. I next introduced the staff, and passed my finger along it into the bladder, by which I was immediately conducted to the stone; but I thought the opening had not been made sufficiently large by the gorget, therefore dilated it by a very slight effort with my finger. The stone being completely exposed, I passed in the forceps again, and took away the calculus without any difficulty. The time occupied by the operation was eight minutes. The boy bore it well.

No untoward symptom occurred afterwards until about the eighth day, when the water returned to its accustomed course, which was attended by severe pain, the boy screaming very loudly at each effort to make water. This continued till the fourteenth day, the wound having appeared foul, and the surrounding parts inflamed, for two or three days previously, when a substance came away from the wound having the following appearance:

It is a cyst, apparently of the same structure as the bladder; its size is sufficient to contain the calculus, which weighed two drachms; the opening into it is just large enough to admit of its exit, and its whole internal surface is lined with calculous matter, in fact, studded with large pieces of calculi.

Since the coming away of the cyst, the wound has continued to improve in appearance daily, and is now (December 27th) nearly healed. The water passes in its natural channel.

*Reflections.*—I have no hesitation in pronouncing the substance voided by the wound to be a cyst, in which the stone was contained previous to the operation. Its appearance, its size, its being lined with calculous matter, and the opening into it being ragged and just large enough to admit of the stone passing out of it, are circumstances decisive of its nature.

By every examination the stone was found to be in the same situation; and by several surgeons in the country, previous to his admission, it had been pronounced that no stone existed.

The existence of the stone in the cyst, by which it was almost wholly covered, produced the embarrassment in the operation.

It is evident that the gorget opened the bladder *sufficiently*, or the stone could not have been taken out. It is also clear that the forceps and finger were *really* passed into the *bladder*, and not, as *feared*, *behind* it; but that they overreached the only part of the stone which was exposed to the cavity of the bladder.

The difficulty in this case was much increased by the youth of the child, and consequently the incomplete development of parts, by which one part could hardly be distinguished from another. The urine also escaped before the introduction of the gorget, which rendered its entrance into the bladder doubtful.

I am anxious to bring this case into notice, because I believe the occurrence to be uncommon, having never met with or heard of a similar case. But I am desirous of bringing it forward at this particular time, because the public seem unwilling to believe that there are difficulties in the operation for the stone; because it is supposed that this operation (concerning which more has in every age been written, and as to the mode of performing it more differences of opinion have existed than in any other operation in surgery,) has now all at once lost all its terrors, both to the patient and operator. In fact, it is considered that no patient need, under any circumstances, be lost from it, and that no operator should exceed a few minutes by his pupil's watch.

The unfortunate case of Mr. Bransby Cooper, which has of late appeared before the public, and has been stigmatised in such disgusting, unmerited, and libellous terms, demands that all should be done to recover it from the imputations cast upon it, not only for the vindication of the operator, whose character stands on the first authority of this country, but for the benefit of all men who are engaged in situations which oblige them to operate before numerous spectators.

The above case was one which fortunately was not protracted; but the same circumstances might have led to its further delay, and even its noncompletion. And again, had not the cyst come away, the difficulties would have remained unexplained, and been attributed to unskilfulness and want of dexterity, as imputed to Mr. B. Cooper.

